# Psychosocial characteristics pattern correlated with HIV-related risky sexual behavior among HIV-negative men who have sex with men: a latent profile analysis

**DOI:** 10.1265/ehpm.22-00157

**Published:** 2023-01-11

**Authors:** Mengxi Zhai, Zhizhou Duan, Jiawei Tian, Qingqing Jiang, Biao Zhu, Chenchang Xiao, Bin Yu, Hong Yan

**Affiliations:** 1 School of Public Health, Wuhan University, Wuhan, Hubei Province, China; 2 Preventive Health Service, Jiangxi Provincial People’s Hospital, The First Affiliated Hospital of Nanchang Medical College, Nanchang, Jiangxi, China; 3 City College, Wuhan University of Science and Technology, Wuhan, China

**Keywords:** HIV-negative MSM, HIV-related risky behaviors, Psychosocial characteristics, Latent profile analysis

## Abstract

**Background:**

Men who have sex with men (MSM) have become a high risk population of HIV infection due to their risky sexual behaviors. The latent pattern of psychosocial characteristics plays an important effect in HIV-related risky behaviors among HIV-negative MSM.

**Method:**

Participants were recruited from Wuhan, Nanchang, and Changsha city from September 2017 to January 2018. Social support was assessed by the multidimensional scale of social support, Connor-Davidson Resilience scale-10 items for reliance, the assessment of Stigma towards Homosexuality for sexual minority stigma, the Likert subscale of nondisclosure for identity concealment, the ACE questionnaire-Kaiser-CDC for adverse childhood experience, the Centers for Epidemiological Studies Depression Scale for depression. Latent profile analysis (LPA) and multivariate regression were used to analyze the data.

**Results:**

Three psychosocial characteristic patterns were revealed by the LPA. “Social support and resilience group” (SR group), “Identity concealment group” (IC group) and “Adverse childhood experience” (ACE group) were identified, respectively. In comparison with “SR group”, “IC group” have a higher likelihood of one-night male partners (AOR = 2.74, 95%CI = [1.54, 4.90]), both fixed and one-night male partners (AOR = 2.01, 95%CI = [1.34, 3.01]) and HIV-unsure male partner (AOR = 2.12, 95%CI = [1.44, 3.13]). Similarly, “ACE group” were more likely having inconsistent condom use (AOR = 2.58, 95%CI = [1.41, 4.73]), and having sex with HIV-positive male partner (AOR = 4.90, 95%CI = [1.95, 12.30]) with comparison of “SR group”. In addition, we further revealed that “ACE group” had a higher ratio (90.0%) of inconsistent condom use among MSM whose male partners were HIV-positive.

**Conclusions:**

Six important psychosocial factors were divided into three latent pattern classes. Compared with “SR group”, “IC group” and “ACE group” were more likely to engage in HIV-related risky sexual behaviors. Further research may pay more attention to “IC group” and “ACE group” for targeted intervention.

## Introduction

Men who have sex with men (MSM) have become a high-risk population of HIV infection in both China and the world [1–3]. Previous studies have revealed that HIV infection has been up to 6.5% among MSM and it has led to a great burden around the world [4–6]. And especially, it was reported that the rate of same-sex sexual intercourse has reached 28.2% in new HIV infections [7]. Similarly, MSM has been the focus population in China due to the large number of MSM and the high rate of new HIV infection. Ying Wang’s study in 2017 reported that there were 10–25 million gay men in China which was only a part of all MSM due to their identity concealment [8]. In addition, the proportion of MSM has increased from 61.7% in 2011 to 73.9% in 2016 among newly identified HIV cases in China [9].

HIV-related risky sexual behaviors were crucial factor in HIV transmission among MSM [10]. A large amount of research indicated that multiple sexual partners, male partner type, and inconsistent condom use can increase the risk of HIV infection [11–13]. Furthermore, HIV status of male sex partners and sex after drinking play an important role in HIV risk assessment [14, 15]. Each Chinese MSM has been reported 6.6 anal sex partners and 7.2 oral sex partners, and the rate of condom use is low (approximately 10%) [16]. Reducing HIV-related risky sexual behaviors was an important step to preventing HIV transmission, highlighting a better understanding of it.

Studies have confirmed that psychosocial factors play an important role in the occurrence of HIV-related risky behaviors [8, 17]. It has been reported that sexual minority stigma, adverse childhood experience (ACE), depression symptoms, identity concealment, social support and resilience are important influencing factors of HIV-related risky behaviors in MSM [18–20]. As a sexual minority, MSM has unique psychosocial characteristics that they face a high level of discrimination [17], and therefore are more inclined to conceal their identity [21]. The increasing research have revealed that sexual minority stigma and identity concealment was positively associated with several of HIV-related risky behaviors, e.g. it was 1.28 times more likely to have unprotected anal sex when MSM had higher levels of sexual concealing and stigmatization [21, 22]. MSM also have more ACEs [23] and depressive symptoms [24]. In Ahaneku’s study, depression was positively correlated with risky sexual behaviors and in turn increased 1.84 times HIV infection risk [25]. In an annual cross-sectional survey, Bertolino and his colleagues found that MSM having adverse childhood experience (ACE) were more likely to self-report condomless anal intercourse, which was 1.13 times than those participants who didn’t experience ACE [23]. However, social support and resilience have been confirmed to be a protective factors against HIV-related risky behaviors possibly by alleviating the level of discrimination and depression in MSM [26, 27].

The influence of a single psychosocial characteristics on risky sexual behavior had been revealed, but many psychosocial factors exist at the same time [28]. It is unclear how the combined effects of multiple psychosocial characteristics act on HIV-related risky sexual behavior, particularly in HIV-negative MSM. Traditional variable-centered analysis failed to reveal the potential information ignoring the heterogeneity and combined effect of psychosocial characteristics. Latent profile analysis (LPA) was a person-centered approach to exploring the particular patterns of the population [29]. Therefore, in this study, we firstly used this method to reveal the latent class patterns of psychosocial characteristics; secondly, we further explored the association between latent patterns of psychosocial characteristics and HIV-related risky sexual behaviors among HIV-negative MSM.

## Materials and methods

### Sample and procedure

With the help of local MSM organizations, participants were recruited by peer recommendation and routine HIV test services from September 2017 to January 2018 in Wuhan, Changsha, and Nanchang, which all were provincial capital of central provinces in China. For more details please see published articles [20, 30, 31]. A total of 800 participants were recruited and 749 completed questionnaires, with a response rate of 93.6%. In this study, the inclusion criteria were: 1) man and were over 16 years old; 2) have sex with man actively in the past six months; 3) self-reported that HIV status was negative; 4) agree to provide informed consent. Of all respondents, 160 were excluded for having no sex with man in the past six months and 589 were included in this statistical analysis.

### Measurements

#### Socio-demographic variables

Basic socio-demographic variables include age, ethnicity (Han and others), educational level (High school or lower and College or higher), marital status (Unmarried and Married/divorced), employment status (Unemployed and Employed), monthly income (<1000 Yuan; 1000–3000 Yuan; 3001–6000 Yuan; >6000 Yuan) and sexual orientation (Homosexual; Bisexual; Unsure/Heterosexual) were collected.

#### Psychosocial variables

Sexual minority stigma was assessed by the assessment of Stigma towards Homosexuality (China Version) [32]. It consists of three subscales, including perceived stigma (Cronbach’s alpha was 0.554, 3 items, e.g., “How often you feel that your homosexual orientation hurts and stigmatizes your family?”), enacted stigma (Cronbach’s alpha was 0.777, 6 items, e.g., “How often does your family refuse to accept you because of your sexual orientation?”), and the other stigma (1 item, “How often you are made fun of because of your sexual orientation?”). The total score ranges from 10 to 40 and a higher sum score indicates higher level of sexual minority stigma. The Chinese version of the scale has been confirmed applicable to the Chinese MSM population, and Cronbach’s alpha was 0.745 in this study.

ACE was measured by the ACE questionnaire developed in the Kaiser-CDC study [33, 34]. Participant reported questions related to their experiences before age 18. It includes 10-item (yes/or) and the total score ranges from 0 to 10 with higher sum score indicating a higher likelihood of ACE. The questionnaire consists of three dimensions, including abuse (Cronbach’s alpha was 0.640, 3 items, e.g., “Are parents or other adults often or very aggressive, pushing you, grabbing you, slapping you, throwing things at you? Or beat you bruised?”), neglect (Cronbach’s alpha was 0.593, 2 items, e.g., “Do you often feel unloved and unimportant at home? Or family members who are not close, caring for or supporting each other?”) and household challenge (Cronbach’s alpha was 0.620, 5 items, e.g., “Does someone in the family suffer from depression or other mental illness?” Or “Do you have a family member who has committed suicide?”). This instrument has been confirmed good reliability and validity in the Chinese population [35, 36], and Cronbach’s alpha was 0.778 in this study.

Depression symptoms were assessed by The Centers for Epidemiological Studies Depression Scale (CESD) [37]. It consists of four dimensions with 20 items, including depressed affect (Cronbach’s alpha was 0.869, 8 items, e.g., “I feel lonely.”), positive affect (Cronbach’s alpha was 0.785, 4 items, e.g., “I sense that the future is promising.”), somatic and retarded activity (Cronbach’s alpha was 0.761, 6 items, e.g., “I can’t concentrate when I’m doing something”), and interpersonal problems (Cronbach’s alpha was 0.780, 2 items, e.g., “I feel like people don’t like me”). The positive affect dimension uses the reverse scoring method. The answers were coded on a 4-points Likert scale from 0 (never) to 3 (always). A higher sum score indicates a severe level of depression symptoms and this scale has shown good reliability and validity in the Chinese population [38, 39]. Cronbach’s alpha was 0.87 in this study.

Identity concealment was assessed by a 6-item 5-points Likert subscale of nondisclosure [40], and each item is scored from 1 (never) to 5 (always) and the total score ranges from 6 to 30. These items include “I made some changes in the way I dressed and spoke because I didn’t want people to think my sexual identity”, “I don’t take homosexual people to social events because I don’t want people to know about my sexual identity”, and so on. Higher sum score indicates a higher likelihood of identity concealment. Cronbach’s alpha was 0.907 in this study.

Social support was measured by the multidimensional scale of perceived social support [41]. It is a 7-points Likert scale including family support (Cronbach’s alpha was 0.868, 4 items, e.g., “My family is willing to assist me in making all kinds of decisions”), friend support (Cronbach’s alpha was 0.915, 4 items, e.g., “I can count on my friends in times of trouble”), and others support (Cronbach’s alpha was 0.865, 4 items, e.g., “There is a special person in my life who cares about my feelings.”). A higher total score indicates a higher level of social support [42]. Cronbach’s alpha was 0.934 in this study.

Resilience was measured by Connor-Davidson Resilience scale-10 items (CD-RISC-10) [43]. The items of the scale contain “I can handle whatever happens.”, “Dealing with stress makes me feel empowered.”, “I will not be discouraged by failure”, and so on. Each item is rating from 1 (never) to 5 (always) with sum scores ranging from 10 to 50 and the higher sum score indicates a higher level of resilience. The scale has been reported a good reliability and validity [44, 45] and Cronbach’s alpha was 0.944 in this study.

#### HIV-related risky sexual behaviors

We measured five risky sexual behaviors in the present study. The first was multiple sexual partner which was measured by asking “How many sexual partners did you have in the past six months?”, and participants having 2 or more male partners were classified as multiple sexual partners; the second was male partner type which was measured by asking participants’ type of sexual partner in the past six months (fixed partners, one-night partners and both of them); the third was sex after drinking, participants chose yes or no based on whether they had sex after drinking in the past six months; the fourth was HIV status of male partner, which was measured by the question “Do you have sex with man whose HIV status was positive in the past six months” and the options included yes, no, or unsure; the fifth is inconsistent condom use, which was assessed by asking the frequency of condom use during having sex with men in the past six months, with options from 0 (never) to 4 (every time). In statistical analysis, 0–3 were classified as inconsistent condom use and 4 were classified as consistent condom use.

### Statistical analysis

#### Latent profile analysis (LPA)

The latent profile analysis is a person-centered approach, aiming to find hidden groups from measurable continuous variables. In addition, it is noteworthy that the pattern of complex identity attributes was used to characterize individuals and further divided into latent classes in LPA. In this study, psychosocial variables (sexual minority stigma, ACE, depression, identity concealment, social support, resilience) were incorporated into the LPA.

To explore the number of latent classes, a combination of model indexes need to be considered as follows: Log-likelihood, Bayesian information criterion (BIC), Adjust Bayesian information criterion (adj. BIC), Akaike information criterion (AIC). Lower Log-likelihood, BIC, adj. BIC and AIC indicated better model fit. Using the Lo-Mendel-Rubinto adjust bootstrap likelihood ratio test (LRT), if the statistical value is less than 0.05, it indicated this number model class (K) was better than the last one (K-1). The entropy index was used to determine the accuracy of latent profile model and higher entropy index showed higher accuracy of model. In order to increase the generalizability of LPA analysis, the minimum percentage of the classes was set at 5% [46]. LPA was conducted by Mplus 7.1 software.

#### Logistic regression model

After determining the potential classes of psychosocial factors, logistic regression model was used to explore the relationship between the latent pattern of psychosocial factors and HIV-related risky sexual behaviors. In detail, multiple binary logistic regression models were performed to explore the associations between latent classes of psychosocial factors and multiple sexual partners/sex after drinking/inconsistent condom use. Multiple logistic analyses was used to explore the associations between latent classes of psychosocial factors and male partner type/HIV status of male partner. In all logistic regression models, HIV-related risky behaviors were dependent variables, the latent pattern of psychosocial factors was independent variable, and socio-demographic variables were adjusted. Logistic regression model was conducted by SPSS 17.0 and statistical significance was set at 0.05 (two-tails) in this study.

## Results

### Characteristic of samples

As shown in Table 1, the majority of participants (94.91%) were Han ethnic with an average of 27.14 (SD = 8.23) years old. Of all participants, 76.06% were in college or higher, 84.38% were unmarried, and 89.47% were employed. Only 19.52% of MSM reported their monthly income was more than 6000 Yuan (925$). Most MSM (72.33%) were homosexual.

**Table 1:** Basic socio-demographic, psychosocial and HIV-related risky sexual behavior characteristics of samples (N = 589).

**Variables**	**Mean (SD)/n (%)/Median (IQR)**
***Basic socio-demographic characteristics***	
**Age**	27.14 (8.23)
**Ethnicity**	
Han group	559 (94.91)
Others	30 (5.09)
**Education level**	
High school or lower	141 (23.94)
College or higher	448 (76.06)
**Marital status**	
Unmarried	497 (84.38)
Married/divorced	92 (15.62)
**Employment status**	
Unemployed	62 (10.53)
Employed	527 (89.47)
**Monthly income (RMB)**	
<1000 Yuan	70 (11.88)
1000–3000 Yuan	213 (36.16)
3001–6000 Yuan	191 (32.43)
>6000 Yuan	115 (19.52)
**Sexual orientation**	
Homosexual	426 (72.33)
Bisexual	117 (19.86)
Unsure/heterosexual	46 (7.81)
***Psychosocial characteristics***	
**Sexual minority stigma**	15.97 (4.46)
**Adverse childhood experience**	1.00 (1.00)
**Depression symptoms**	17.77 (10.41)
**Identity concealment**	17.18 (6.68)
**Social support**	60.53 (12.51)
**Resilience**	36.57 (8.44)
***HIV-related risky sexual behavior characteristics***	
**Multiple sexual partner**	
Yes	310 (52.63)
No	279 (47.37)
**Partner type**	
All were fixed partners,	315 (53.48)
All were one-night partners	73 (12.39)
Both of them	201 (34.13)
**Sex after drinking**	
Yes	183 (31.18)
No	404 (68.82)
**HIV status of male partner**	
Positive	36 (6.11)
Negative	347 (58.91)
Unsure	206 (34.97)
**Inconsistent condom use**	
Yes	271 (49.72)
No	274 (50.28)

The average score of sexual minority stigma was 15.97 (SD = 4.46) among MSM. On average, participants reported 1 (IQR = 1.00) ACE before the age of 18. The average scores of depression symptoms and identity concealment were 17.77 (SD = 10.41) and 17.18 (SD = 6.68), respectively. The social support and resilience scores were 60.53 (SD = 12.51) and 36.57 (SD = 8.44).

More than half of the participants (52.63%) had multiple sexual partners in the past six months. 34.13% participants had fixed and one-night partners and 12.39% participants’ partners were all one-night partners. Nearly a third of MSM (31.18%) reported having drunk sex in the past six months. 6.11% participants had HIV-positive partner and 34.97% participants don’t know the HIV status of partner. Only 50.28% of MSM used condoms all the time they had sex.

### LPA of psychosocial characteristics

**Fig. 1:**
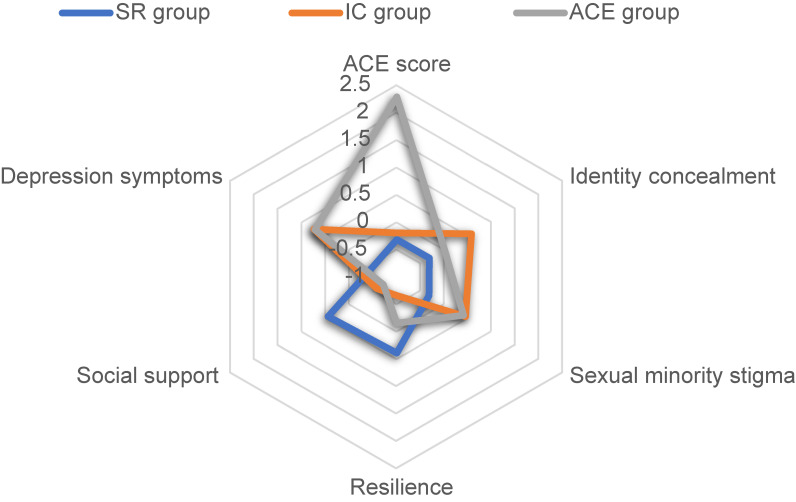
Latent classes by categorical Z-score mean.

The number of latent classes ranging from 1 class model to 4 class model was tested (see Table 2). LRT test confirmed that 3 class model was better than 2 class model, and 2 class model was better than 1 class model. However, 4 class model didn’t show significantly better than 3 class model. And AIC index, BIC index, and Log-likelihood index showed a lower amount of reduction after 3 class model. Therefore, 3 class model was the best in this study. In addition, we use Z-scores of psychosocial factors to compare with each other due to different measures. Figure 1 further showed the Z-scores of each psychosocial factor.

**Table 2:** Latent class fit indices.

	**Log-likelihood**	**Entropy**	**AIC**	**BIC**	**Adj. BIC**	**The minimum percentage ** **of the classes**	**Adj. LRT ** **P-value**
1 class model	-	-	22663.92	22716.46	22678.36	-	-
2 class model	−11168.64	0.68	22375.27	22458.46	22398.14	30.73%	0.001
3 class model	−11070.71	0.74	22193.41	22307.25	22224.71	10.70%	0.015
4 class model	−10999.46	0.78	22064.91	22209.40	22104.64	2.37%	0.173

Note: AIC = Akaike information criteria BIC = Bayesian information criteria LRT = Lo-Mendell-Rubin test

Class 1, “social support and resilience group” (SR group), was consisted of 340 (57.72%) individuals, social support was the highest in all psychosocial factors, resilience had the second highest score; ACE score, identity concealment, sexual minority stigma, and depression ranked the lowest in three model classes.

Class 2, “identity concealment group” (IC group), consisted of 186 (31.58%) individuals, and identity concealment was the highest point in all psychosocial factors, depression was the second highest score. In all three classes, sexual minority stigma and depression ranked 1, ACE and social support score ranked 2, resilience ranked 3.

Class 3, “ACE group”, with consistent of 63 (10.70%) individuals and ACE score was the highest point in all psychosocial factors, depression ranked 2. In all three classes, identity concealment, sexual minority stigma, resilience and depression ranked 2, social support ranked 3.

Table 3 shows the basic socio-demographic, psychosocial and HIV-related risky sexual behavior characteristics of three groups. There was no significant difference in demographic characteristics among three groups. However, all psychosocial characteristics were different in three groups. In terms of HIV-related risky sexual behavior, the significant differences in partner type, HIV status of male partner and inconsistent condom use among three groups were observed.

**Table 3:** Basic socio-demographic, psychosocial and HIV-related risky sexual behavior characteristics of three groups. (N = 589).

**Variables**	**SR group**	**IC group**	**ACE group**	**χ^2^/F/H**
***Basic socio-demographic characteristics***				
**Age, Mean (SD)**	27.22 (8.20)	26.97 (7.80)	27.25 (9.62)	0.059
**Ethnicity, n (%)**				4.909
Han group	317 (93.24)	180 (96.77)	62 (98.41)	
Others	23 (3.90)	6 (3.23)	1 (3.33)	
**Education level, n (%)**				0.169
High school or lower	81 (23.82)	46 (24.72)	14 (22.22)	
College or higher	259 (76.18)	140 (75.27)	49 (77.78)	
**Marital status, n (%)**				1.019
Unmarried	284 (83.53)	161 (86.56)	52 (82.54)	
Married/divorced	56 (16.47)	25 (13.44)	11 (17.46)	
**Employment status, n (%)**				1.807
Unemployed	33 (9.71)	24 (12.90)	5 (7.94)	
Employed	307 (90.29)	162 (87.10)	58 (92.06)	
**Monthly income (RMB), n (%)**				4.142
<1000 Yuan	38 (11.18)	20 (10.75)	12 (19.05)	
1000–3000 Yuan	121 (35.59)	70 (37.63)	22 (34.92)	
3001–6000 Yuan	112 (32.94)	62 (33.33)	17 (26.98)	
>6000 Yuan	69 (20.29)	34 (18.28)	12 (19.05)	
**Sexual orientation, n (%)**				4.657
Homosexual	251 (73.82)	132 (70.97)	43 (68.25)	
Bisexual	69 (20.29)	34 (18.28)	14 (22.22)	
Unsure/heterosexual	20 (5.88)	20 (10.75)	6 (9.52)	
***Psychosocial characteristics***				
**Sexual minority stigma, Mean (SD)**	14.55 (3.01)	17.97 (5.21)	17.78 (5.65)	47.495***
**Adverse childhood experience, Median (IQR)**	0.00 (1.00)	1.00 (1.00)	4.00 (2.00)	199.146***
**Depression symptoms, Mean (SD)**	12.13 (7.07)	25.54 (8.16)	25.32 (11.94)	197.213***
**Identity concealment, Mean (SD)**	15.08 (6.52)	21.01 (5.36)	17.22 (5.98)	56.296***
**Social support, Mean (SD)**	66.15 (8.99)	53.30 (11.65)	51.56 (15.00)	112.561***
**Resilience, Mean (SD)**	39.86 (7.43)	31.00 (7.35)	35.24 (7.61)	86.783***
***HIV-related risky sexual behavior characteristics***				
**Multiple sexual partner, n (%)**				0.495
Yes	175 (51.47)	100 (53.76)	35 (55.56)	
No	165 (48.53)	86 (46.24)	28 (44.44)	
**Partner type, n (%)**				16.646**
All were fixed partners,	202 (59.41)	78 (41.94)	35 (55.56)	
All were one-night partners	32 (9.41)	32 (17.20)	9 (14.29)	
Both of them	106 (31.18)	76 (40.86)	19 (30.16)	
**Sex after drinking, n (%)**				1.062
Yes	106 (31.27)	61 (32.80)	16 (25.81)	
No	233 (68.73)	125 (67.20)	46 (74.19)	
**HIV status of male partner, n (%)**				26.266***
Positive	16 (4.71)	10 (5.38)	10 (15.87)	
Negative	223 (65.59)	91 (48.92)	33 (52.38)	
Unsure	101 (29.71)	85 (45.70)	20 (31.75)	
**Inconsistent condom use, n (%)**				9.144*
Yes	146 (46.35)	85 (49.71)	40 (67.80)	
No	169 (53.65)	86 (50.29)	19 (32.20)	

Note: Chi-square test was used for categorical variables, analysis of variance was used for continuous variables and ACE used Kruskal-Wallis method of rank sum test. *p < 0.05; **p < 0.01; ***p < 0.001.

### Logistics regression model of associations between psychosocial characteristic patterns and HIV-related risky behaviors

**Fig. 2:**
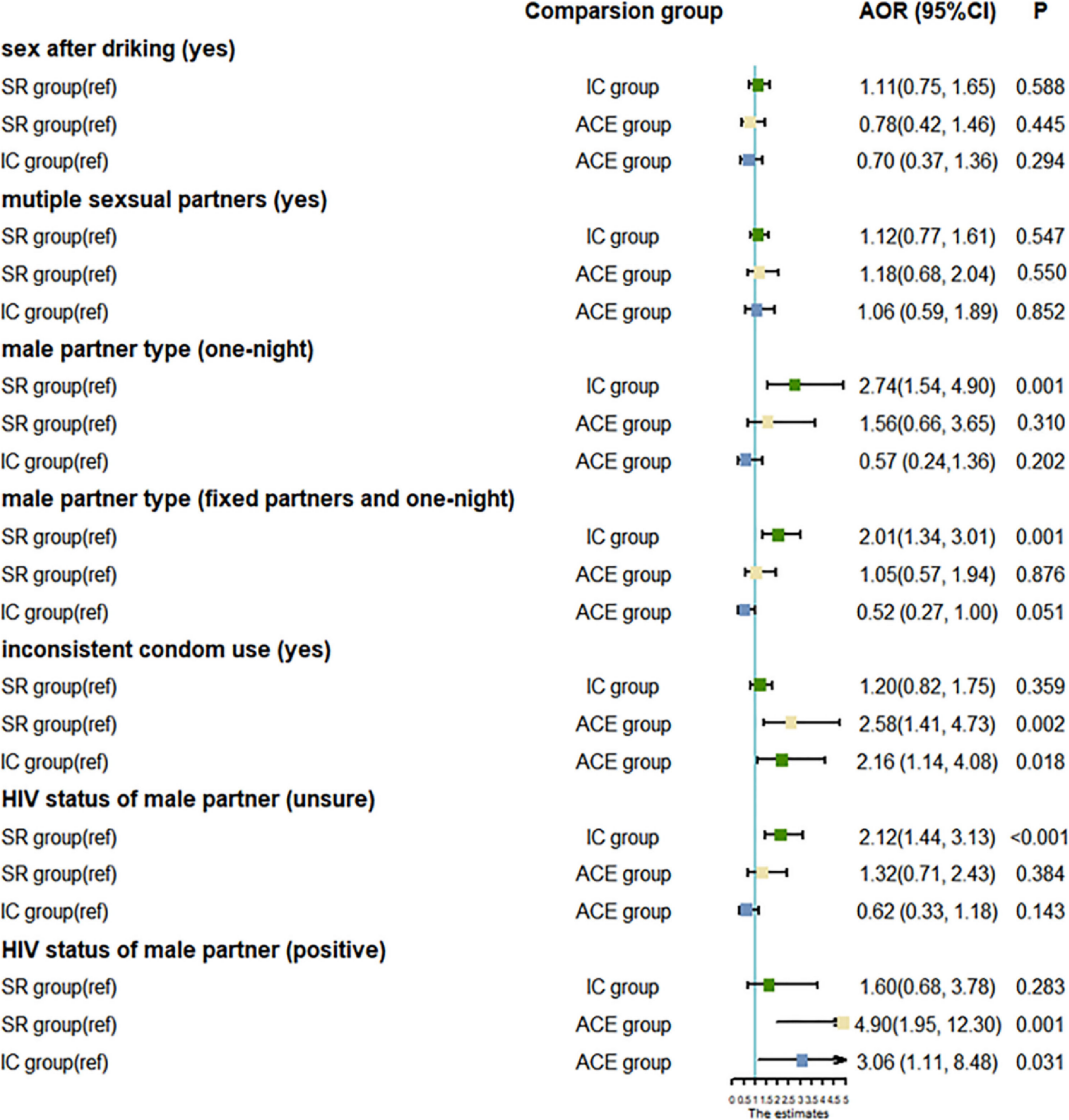
The association between psychosocial pattern and HIV-related risky sexual behaviors.

As shown in Fig. 2, after controlling socio-demographic variables, logistic regression model revealed that IC group were more likely having one-night partner and fixed/one-night (all were one-night partners: AOR = 2.74, 95%CI = [1.54, 4.90]; fixed/one-night partner: AOR = 2.01, 95%CI = [1.34, 3.01]), having sex with male partner with unsure HIV status (AOR = 2.12, 95%CI = [1.44, 3.13]). ACE group were more likely having inconsistent condom use (AOR = 2.58, 95%CI = [1.41, 4.73]), and having sex with HIV-positive male partner (AOR = 4.90, 95%CI = [1.95, 12.30]). Comparing with IC group, ACE group were more likely having inconsistent condom use (AOR = 2.16, 95%CI = [1.14, 4.08]), and having sex with HIV-positive male partner (AOR = 3.06, 95%CI = [1.11, 8.48]).

**Fig. 3:**
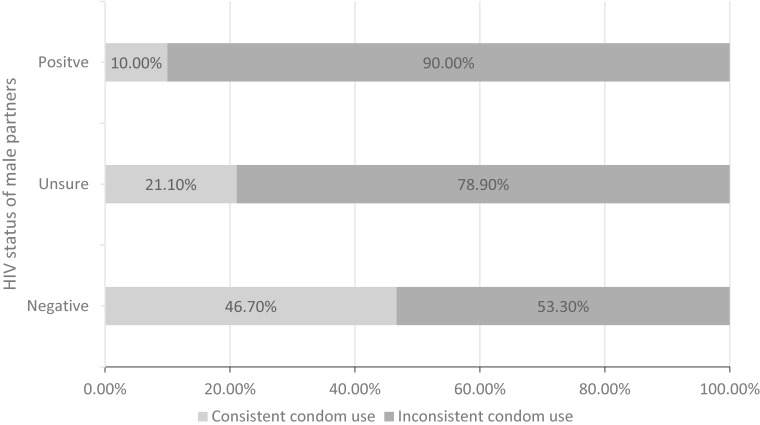
Inconsistent condom use rate of different HIV status of male partner (*χ*^2^ = 6.21, P = 0.045)

Moreover, in Fig. 3, we further found that, compared with MSM having sex with the HIV-negative male partners, those having sex with HIV-positive or HIV-unsure male partners were more likely to inconsistently use condoms in ACE group (*χ*^2^ = 6.21, P = 0.045). However, it was not significant in other group’s comparisons.

## Discussion

This study revealed that six observable psychosocial characteristics were divided into three latent patterns (social support and resilience group, identity concealment group, and ACE group) among HIV-negative MSM. “Social support and resilience (SR) group” showed positive social support and resilience, while the other two groups were contrary. “Identity concealment (IC) group” had high points in identity concealment and depression, in addition, the sexual minority stigma score was not very low in this group. ACE group showed the highest ACE score, and the depression scores and sexual minority stigma score were also high. This aligned well with previous studies [20, 47, 48], showing that multiple psychosocial characteristics can coexist in one person and might influence each other.

Compared with “SR group”, “IC group” was more likely to have one-night male partner, and have sex with HIV-unsure male partner. Studies have shown that stigma can promote identity concealment and depression [20, 47, 49], therefore, it is possible to explain why the IC group had higher scores of identity concealment and depression, and sexual minority stigma was not low. A current study has found that MSM concealing their identity had more occasional partners and were less likely to disclose their HIV status to their partners, which was consistent with our findings [21]. “IC group” had higher sexual orientation concealment scores, indicating that they were reluctant to disclose their information to their friends and acquaintances. They may seek one-night male partners so that they can avoid revealing their sexual orientation to friends and acquaintances. In addition, MSM with identity concealment are inclined to disclose their other information including HIV status [21], and also possibly ignore their casual partners’ HIV status. Unaware of sex partners’ HIV status was an important barrier to reducing the transmission of HIV infection [14, 50]. It has been shown that HIV status of male sex partner plays an important role in HIV risk assessment, especially having sex with HIV-unsure male partner might have a greater risk [51]. In HIV intervention targeted at MSM in the “IC group”, psychological counseling need to be emphasized, and HIV risk awareness education is critical for promoting MSM to proactively understand HIV status of sexual partners and consistently use condoms.

“ACE group” were more likely to have inconsistent condom use and HIV-positive male partner. Previous studies [52–54] have shown that MSM with higher level of ACE exposure was more likely to feel psychological distress (e.g., depression), and in turn pay less attention to HIV infection, increasing the risk of infecting HIV. And it has been revealed that ACE exposure was associated with condomless anal intercourse [23], which was consistent with our findings. It was also found that “ACE group” were more likely to have HIV-positive male partners, and further analysis showed that 90% of MSM in this subgroup reporting ever having sex with HIV-positive partner inconsistently used condoms. This suggested that for MSM in this subgroup, HIV intervention need focus on consistent condom use attitude and behavior.

However, in multiple sexual partners and post-drinking sexual behavior, the significant difference between the three groups wasn’t found. The scores of social support and resilience in the study population were relatively high, which may neutralize the influence of negative psychosocial characteristics on multiple sexual partners and post-drinking behavior to some extent [26, 27]. In addition, multiple sexual partners are common among MSM [55], multiple partners were defined as two or more partners in this study, due to the small sample size, the number of sexual partners was not further stratified, which may also lead to the fact that we did not find a difference in multiple sexual partners behavior among three patterns. The drinking rate (31.41%, sex after drinking alcohol rate was 31.18%) of the participants in this study was not very high, which may affect the results of their sexual behaviors after drinking to a certain extent. This finding also reminds us that psychosocial factors have combined effects on HIV-related risky sexual behaviors, and the influence of various psychosocial characteristics should be considered comprehensively.

Though we used a unique approach to explore HIV-related risky sexual behavior among MSM, there were some limitations in this study. First, this was a cross-sectional study, and causal relationships between psychosocial factors and HIV risk behaviors cannot be determined. Second, the study subjects were obtained through non-random sampling, so there may be selection bias. Finally, we only chose several psychosocial characteristics based on previous studies, other psychosocial variables (e.g., anxiety) were not included in the latent class model, and maybe change the latent profile.

## Conclusion

Basing six important psychosocial factors of depression, resilience, social support, adverse childhood experience, sexual minority stigma, and identity concealment, MSM were divided into three latent pattern classes. Compared with “social support and reliance group”, “identity concealment group” and “ACE group” were more likely to engage in HIV-related risky sexual behaviors (e.g., inconsistent condom use). In HIV intervention targeted at MSM, psychosocial characteristics need to be focused on, especially paying more attention to MSM with higher level of identity concealment or ACE. Besides mental health guidance, promoting serological disclosure of sexual partners and consistent condom use behavior is critical for MSM with higher level of identity concealment or ACE.
